# The Effect of *Msh2* Knockdown on Toxicity Induced by *tert*-Butyl-hydroperoxide, Potassium Bromate, and Hydrogen Peroxide in Base Excision Repair Proficient and Deficient Cells

**DOI:** 10.1155/2013/152909

**Published:** 2013-08-04

**Authors:** N. Cooley, R. H. Elder, A. C. Povey

**Affiliations:** ^1^Centre for Occupational and Environmental Health, Institute of Population Health, Faculty of Medical and Human Sciences, University of Manchester, Manchester M13 9PT, UK; ^2^School of Environment and Life Sciences, Cockcroft Building, University of Salford, Salford M5 4WT, UK

## Abstract

The DNA mismatch repair (MMR) and base excision repair (BER) systems are important determinants of cellular toxicity following exposure to agents that cause oxidative DNA damage. To examine the interactions between these different repair systems, we examined whether toxicity, induced by *t*-BOOH and KBrO_3_, differs in BER proficient (*Mpg* ^+/+^, *Nth1* ^+/+^) and deficient (*Mpg* ^−/−^, *Nth1* ^−/−^) mouse embryonic fibroblasts (MEFs) following *Msh2* knockdown of between 79 and 88% using an shRNA expression vector. *Msh2* knockdown in *Nth1* ^+/+^ cells had no effect on *t*-BOOH and KBrO_3_ induced toxicity as assessed by an MTT assay; knockdown in *Nth1* ^−/−^ cells resulted in increased resistance to *t*-BOOH and KBrO_3_, a result consistent with Nth1 removing oxidised pyrimidines. *Msh2* knockdown in *Mpg* ^+/+^ cells had no effect on *t*-BOOH toxicity but increased resistance to KBrO_3_; in *Mpg* ^−/−^ cells, *Msh2* knockdown increased cellular sensitivity to KBrO_3_ but increased resistance to t-BOOH, suggesting a role for *Mpg* in removing DNA damage induced by these agents. MSH2 dependent and independent pathways then determine cellular toxicity induced by oxidising agents. A complex interaction between MMR and BER repair systems, that is, exposure dependent, also exists to determine cellular toxicity.

## 1. Introduction

Reactive oxygen species (ROS) can induce a wide range of DNA base lesions [1], and different ROS can modify DNA in different ways [2]. Furthermore, given that the biological potency of only a minority of these lesions has been characterised, the precise role, if any, of an individual lesion in inducing toxicity can be unclear. 8-oxoguanine (8-oxoG) is one of the most studied lesions, being formed in high amounts in DNA by ROS, and is potentially mutagenic [3]. 8-oxoG is formed upon exposure to a wide range of agents including hydrogen peroxide (H_2_O_2_; [4]), *tert*-butyl-hydroperoxide (*t-*BOOH; [5]), and potassium bromate (KBrO_3_; [6]), though other DNA base modifications will also be formed [6, 7]. 

ROS induced damage can be repaired by a number of different DNA repair systems including those involving base excision repair (BER; [8]) and mismatch repair (MMR: [9]). BER of oxidative base damage is initiated by the action of a number of different DNA glycosylases that can excise a spectrum of different oxidised DNA lesions. NTH1, for example, removes oxidised pyrimidines as well as 2,6-diamino-4-hydroxy-5-formamidopyrimidine (FapyG) and 4,6-diamino-5-formamidopyrimidine (FapyA: [10, 11]). Loss of hNTH1 increases levels of FapyG and FapyA [11] as well as sensitivity to *γ*-rays and H_2_O_2_ [12]. In human cells, MMR is initiated through the binding of a heterodimer of hMSH2 with either hMSH6 (hMutS*α*) or hMSH3 (hMutS*β*) to the site of the mismatch or base insertion/deletion [13]. Loss of hMSH2 results in an increase in steady state levels of 8-oxoG [14, 15] and increased levels following exposure to H_2_O_2_ [14, 16], methotrexate [17], or ionising radiation [18]. Loss of hMSH2 can also increase other forms of oxidative damage such as 8-oxoadenine and thymine glycol or clustered DNA base lesions following treatment with ionising radiation [18, 19]. 

Though, *Msh2* ^−/−^ cells can show a strong mutator phenotype (e.g., at the *hprt *gene [20]) and have an increased mutation frequency after treatment, for example, with ionising radiation [18], the effects of MSH2 deficiency appear to be cell specific. Thus, there was no evidence of an increased mutation rate or microsatellite instability in *Msh2* ^−/−^ murine embryonic fibroblast (MEF) clones overexpressing hNOX1, despite an increased level of 8-oxodG [16]. Initially, it was also reported that *Msh2* ^−/−^ cells have an increased resistance to the toxic effects of ionising radiation [18, 19] especially at low doses [18], though subsequent work suggests that MMR is not involved in ionising radiation induced cell death [21]. MMR sensitisation may arise through futile cycling of DNA damage or indirectly by generating signals that drive cell fate pathways [18]. However, increased resistance following MSH2 loss is not universally described as it has been reported that methotrexate and H_2_O_2_ treatment resulted in increased sensitivity in clonogenic assays using tumour cells lacking MSH2 [17]. 

Hence, there are a number of unresolved issues regarding the role of MSH2 in removing oxidative DNA damage. Firstly, the effects of MSH2 deficiency appear cell specific for as yet unknown reasons but could include the relative levels of other DNA repair proteins involved in removing oxidative DNA damage. Secondly, although it has been reported that MSH2 has broad substrate specificity, there is a relative lack of information regarding the effects of MSH2 deficiency in cells treated with differing forms of oxidative stress. To address these issues, we have investigated the effects of MSH2 on toxicity induced by KBrO_3_ and *t*-BOOH, in cells proficient and deficient in BER proteins that either remove oxidative DNA damage (NTH1) or alkylated DNA or DNA damaged by lipid peroxidation products (MPG).

## 2. Methods

### 2.1. Cells

BER proficient (*Mpg* ^+/+^, *Nth1* ^+/+^) and deficient MEF cell lines (*Mpg* ^−/−^,* Nth1* ^−/−^) were used as the parental cells for gene silencing [22]. All cells were grown in DMEM-F12 supplemented with 10% fetal bovine serum, 2 mM L-glutamine, and 0.12% sodium bicarbonate, at 37°C in a 5% CO_2_ and 3% O_2_ humidified atmosphere. Stable *Msh2* knockdown cell lines were generated by transfection of the parental cell lines with an shRNA expression vector (pshRNA) containing oligonucleotide inserts specifically designed to knockdown *Msh2* expression. These were 5′-GATGAACTTTGAGTCTTTCG-3′ (*Msh2* ^283^,[22]). A pshRNA vector containing no target sequence was used as a control.

### 2.2. Determination of *Msh2* Expression Levels

*Msh2* expression levels in individual clones were determined by quantitative real-time PCR using a standard curve generated using varying amounts of cDNA from a nontransfected, parental cell line [22]. Total RNA was extracted from confluent cell pellets using an RNeasy Mini Kit (Qiagen), purified by treatment with RNase-free DNase (Promega), and first strand cDNA was synthesised using AMV-reverse transcriptase (Promega). Oligonucleotide sequences used were 5′-TCTTCTTCTGGTTCGCCAGT-3′ (*Msh2* forward primer), 5′-TGATCATTCTCGGGGAACTC-3′ (*Msh2 *reverse primer); 5′-AACTTTGGCATTGTGGAAGG-3′ (*Gapdh* forward primer), 5′-ACACATTGGGGGTAGGAACA-3′ (*Gapdh* reverse primer); *Actin* forward primer, 5′-TGTTACCAACTGGGACGACA-3′, *Actin* reverse primer: 5′-GGGGTGTTGAAGGTCTCAAA-3′. The real-time PCR reaction was performed using the following protocol: 1 cycle of 95°C for 10 min, 40 cycles of 95°C for 15 s, 57°C for 15 s, and 72°C for 30 s followed by a fluorescence reading and 1 cycle of 72°C for 1 min. Finally, a fluorescence reading was taken every 0.2°C between 75°C and 92°C to ensure the presence of a single PCR product. *Msh2* expression in each sample was normalized to the housekeeping gene (Gapdh or actin) expression. Finally, the fold change in the target gene of the sample was expressed as a ratio compared to the target gene expression in the empty vector control.

### 2.3. Western Blot Analysis of MSH2 Protein Levels

Western blot analysis was carried out as described previously [22]. Briefly, proteins obtained from extracts of the cell lines were separated by SDS-PAGE, electrophoretically transferred to nitrocellulose membranes and the membranes blocked with nonfat milk (Marvel) prior to incubation with anti-MSH2 (1 : 2000 dilution, Abcam). HRP-conjugated secondary antibodies (Dako) and the SuperSignal West Dura detection system (Pierce) were used to detect proteins of interest. To ensure equal loading of proteins, the membrane was reprobed with a primary antibody (1 : 4000) against a housekeeping gene or loading control (Gapdh, actin, or *α*-tubulin; Abcam). 

### 2.4. MTT Cytotoxicity Assay

MEFs (typically 500 cells) were plated in a 96-well tissue culture plate, treated with *t*-BOOH (0.1 mM) and KBrO_3_ (0–2 mM) for 72 h, and then 3-(4,5-dimethylthiazol-2-yl)-2,5-diphenyltetrazolium bromide (MTT) was added, and incubation continued for a further 3 h [22]. The medium was removed, and the cells were lysed with DMSO. Formazan formation was determined by absorbance at 590 nm, and the background correction was measured at 690 nm. The number of surviving cells at each concentration was calculated as a percentage of the absorbance from untreated control cells. Results are presented as means ± SEM. 

### 2.5. Clonogenic Survival Assay

The clonogenic survival assay was carried out as described previously [22]. Briefly MEFs (typically 250 cells/well) were plated in a 6-well tissue culture plate, treated every 3-4 days with *t-*BOOH (0–15 *μ*M) or KBrO_3_ (0–250 *μ*M) for 2-3 weeks until cell colonies of greater than 50 cells could be seen in the control wells. After staining with crystal violet, cell colonies (nucleus of >50 cells) were counted under a microscope. Clonogenic survival was calculated as the number of colonies observed after treatment divided by the number of cells initially plated after adjustment for the survival fraction (viz., the number of colonies observed without treatment divided by the number of cells plated). Results are presented as means ± SEM. 

### 2.6. Statistical Analysis

Data was analyzed by repeated measures ANOVA using STATA 8. Linear regression was used to examine the relationship between treatment and cell line with the untreated cell line transfected with pshRNAEmpty used as the reference. The date the experiment was performed was included in the linear regression model. 

## 3. Results 

### 3.1. *Msh2* Expression

**Figure 1:**
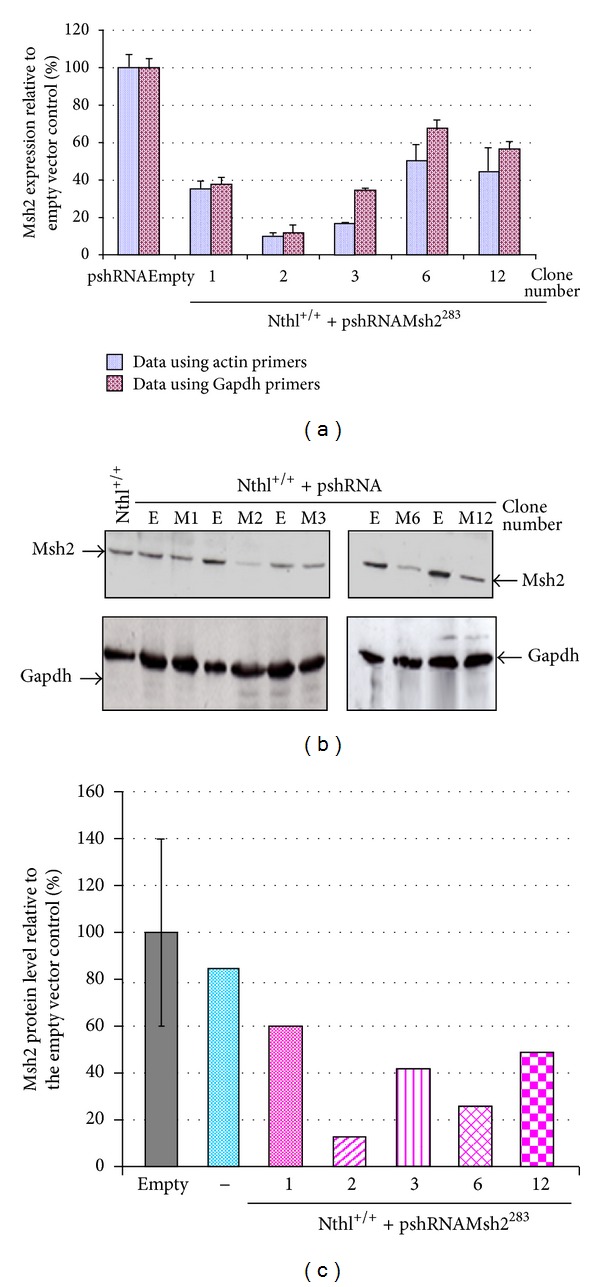
*Msh2 *silencing in *Nth1* ^+/+^ cells. (a) Real-time data analysis for *Msh2 *gene expression in *Nth1* ^+/+^ clones identified by screening. The data is an average of two real-time PCR reactions (each in triplicate) and analyzed using either *Actin *or *Gapdh *as the reference gene. (b) Western blot analysis for MSH2 and Gapdh protein expression using whole cell extracts. E is the empty vector control and M1, M2, M3, M6, and M12 are different clones. (c) Quantification of the Western blot band intensity and the data expressed as a percentage of the pshRNAEmpty cell extract. The second blue column marked (−) is *Nth1* ^+/+^ cells.

The pshRNA*Msh2283* vector was used to transfect MEFs, and a reduction in *Msh2* expression was observed, using either *Gapdh* or *Actin* as the reference genes when compared to the empty vector control as shown in Figure 1 for *Nth1* ^+/+^ MEFs. Different clones were isolated from *Mpg* ^+/+^ *, Mpg* ^−/−^, *Nth1* ^+/+^, and *Nth1* ^−/−^ MEFs, and cell lines chosen for the cytotoxic assays (*Mpg* ^+/+^ clone 9*, Mpg* ^−/−^ clone 1* Nth1* ^+/+^ clone 2, and *Nth1* ^−/−^ clone 1) all had high levels of MSH2 knockdown as estimated by either real-time PCR (mean 84 ± 4% range 79–88%) or western analysis (mean 76 ± 4% range 73–80%).

### 3.2. Treatment with *t*-BOOH

**Figure 2:**
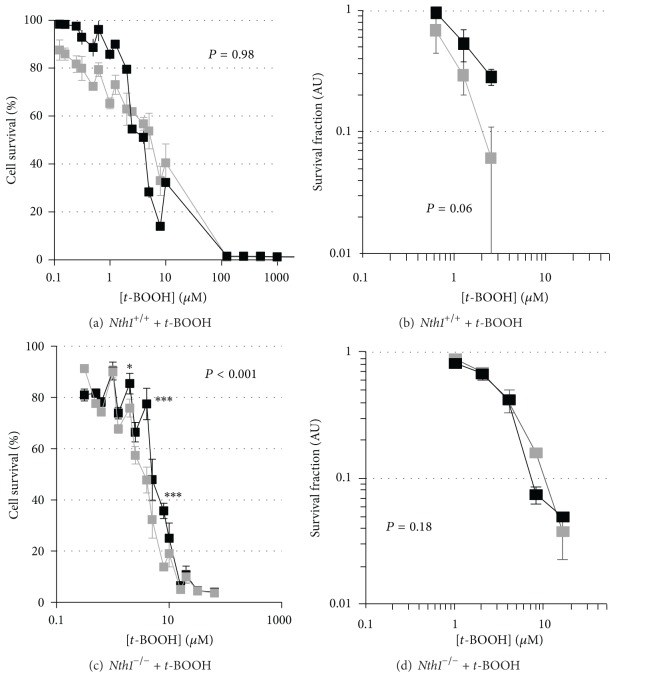
MTT and clonogenic survival curves for *t*-BOOH treatment of *Nth1* ^+/+^ and *Nth1* ^−/−^ MEFs with and without reduced *Msh2* expression. MTT (a) and clonogenic survival (b) curves for *Nth1* ^+/+^ + pshRNA*Msh2283*, clone 2 (88%* Msh2 *gene silencing; black square) and *Nth1* ^+/+^ + pshRNA empty (grey square) treated with *t*-BOOH. MTT (c) and clonogenic survival (d) curves for *Nth1* ^−/−^ + pshRNA*Msh2283*, clone 1 (85% *Msh2 *gene silencing; solid square), or *Nth1* ^−/−^ + pshRNA empty (grey square) treated with *t*-BOOH. Results are expressed as mean ± SEM; *P < 0.05, **P < 0.01, and ***P < 0.001.

*Msh2* knockdown in *Nth1* ^+/+^ cells had little effect on cellular resistance to *t-*BOOH as assessed by the MTT assay (Figure 2(a)), but there was evidence of increased resistance using the clonogenic assay (Figure 2(b)). In *Nth1* ^−/−^ MEFs, *Msh2 *knockdown increased resistance as assessed by the MTT assay (Figure 2(c)) but not the clonogenic assay (Figure 2(d)). 

**Figure 3:**
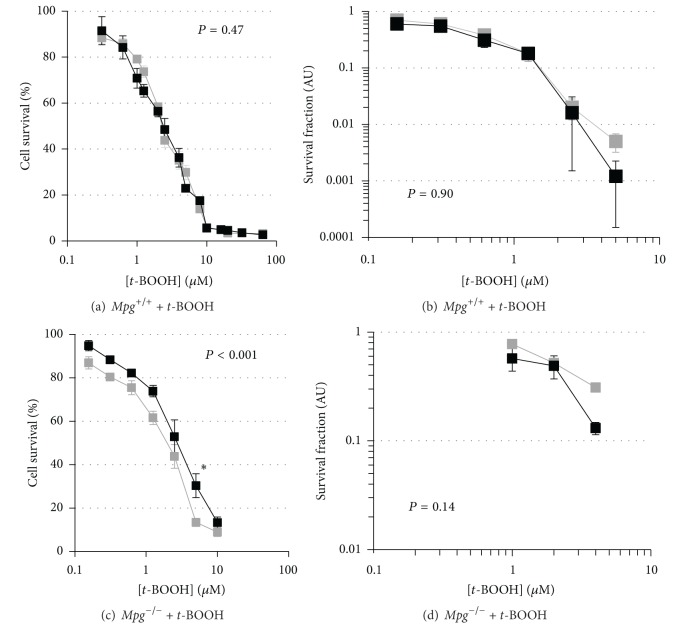
MTT and clonogenic survival curves for *t*-BOOH treatment of *Mpg* ^+/+^ and *Mpg* ^−/−^ MEFs with and without reduced *Msh2* expression. MTT (a) and clonogenic survival (b) curves for *Mpg* ^+/+^ + pshRNA*Msh2283*, clone 9 (85% *Msh2 *gene silencing; black square), or *Mpg* ^+/+^ + pshRNA empty (grey square) cells treated with *t*-BOOH. MTT (c) and clonogenic survival (d) curves for *Mpg* ^−/−^ + pshRNA*Msh2283*, clone 1 (79%* Msh2 *gene silencing; solid square), or *Mpg* ^−/−^ + pshRNA empty (grey square) cells treated with *t*-BOOH. Results are expressed as mean ± SEM; *P < 0.05, **P < 0.01, and ***P < 0.001.

Reduction in *Msh2* expression in *Mpg* ^+/+^ cells had little on cellular sensitivity to *t*-BOOH using either the MTT (Figure 3(a)) or clonogenic assay (Figure 3(b)), whereas in* Mpg* ^−/−^ cells, *Msh2* knockdown increased resistance in the MTT assay (Figure 3(c)) but not the clonogenic assay (Figure 3(d)). 

### 3.3. Treatment with KBrO_3_

**Figure 4:**
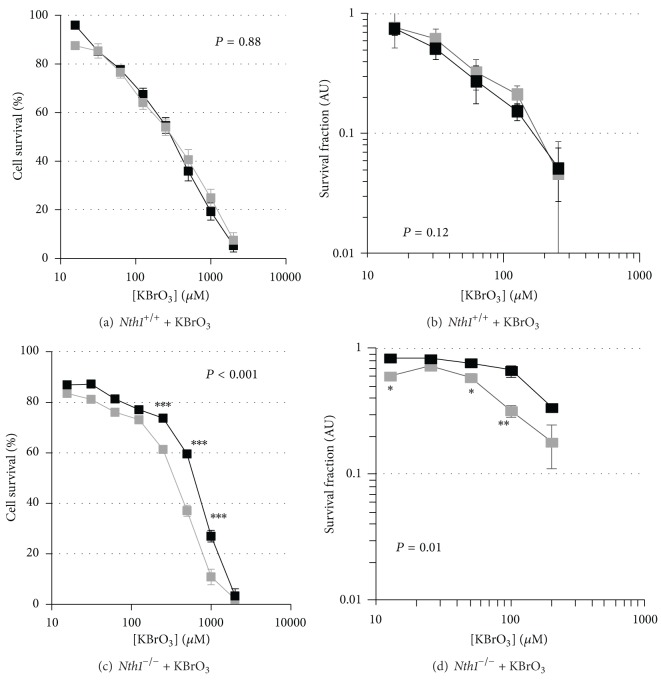
MTT and clonogenic survival curves for KBrO_3_ treatment of *Nth1* ^+/+^ and *Nth1* ^−/−^ MEFs with and without reduced *Msh2* expression. MTT (a) and clonogenic survival (b) curves for *Nth1* ^+/+^ + pshRNA*Msh2283*, clone 2 (88%* Msh2 *gene silencing; black square), and *Nth1* ^+/+^ + pshRNA empty (grey square) treated with KBrO_3_. MTT (c) and clonogenic survival (d) curves for *Nth1* ^−/−^ + pshRNA*Msh2283*, clone 1 (85% *Msh2 *gene silencing; solid square), or *Nth1* ^−/−^ + pshRNA empty (grey square) treated with KBrO_3_.

**Figure 5:**
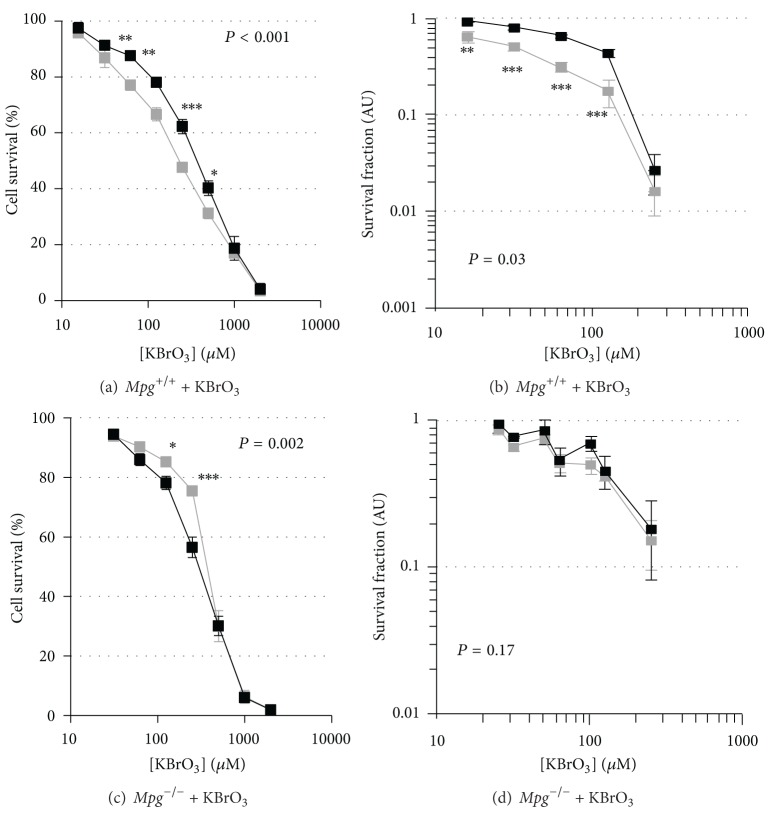
MTT and clonogenic survival curves for KBrO_3_ treatment of *Mpg* ^+/+^ and *Mpg* ^−/−^ MEFs with and without reduced *Msh2* expression. MTT (a) and clonogenic survival (b) curves for *Mpg* ^+/+^ + pshRNA*Msh2283*, clone 9 (85% *Msh2 *gene silencing; black square), or *Mpg* ^+/+^ + pshRNA empty (grey square) cells treated with KBrO_3_. MTT (c) and clonogenic survival (d) curves for *Mpg* ^−/−^ + pshRNA*Msh2283*, clone 1 (79%* Msh2 *gene silencing; solid square), or *Mpg* ^−/−^ + pshRNA empty (grey square) cells treated with KBrO_3_. Results are expressed as mean ± SEM; *P < 0.05, **P < 0.01, and ***P < 0.001.

Reduction in *Msh2 *gene expression in *Nth1* ^+/+^ MEFs did not alter KBrO_3_ toxicity (Figures 4(a) and 4(b)) but resulted in increased resistance in *Nth1* ^−/−^ MEFs using both the MTT (Figure 4(c)) and clonogenic (Figure 4(d)) assays. Decreased MSH2 expression in *Mpg* ^+/+^ cells resulted in increased resistance as assessed using the MTT (Figure 5(a)) and the clonogenic assay (Figure 5(b)) whereas such reduction decreased resistance in *Mpg* ^−/−^ cells using the MTT assay (Figure 5(c)) but not the clonogenic assay (Figure 5(d)). 

## 4. Discussion

Results of this study reveal a complex interaction between oxidative DNA damage, MSH2 function, and the activity of NTH1 and MPG that helps to determine cellular toxicity. Interestingly, results suggest that, whilst the presence or absence of NTH1 activity can influence MSH2 dependent toxicity induced by ROS (which is predictable as NTH1 removes oxidative DNA base lesions [10]), loss of MPG activity also reveals toxicity, that is, MSH2, and exposure dependent implying MSH2 also acts upon MPG substrate lesions. Loss of MSH2 typically results in increased resistance to DNA damaging agents, and our results are in general consistent with this model (Table 1). However, loss of MSH2 in *Mpg* ^−/−^ MEFs resulted in increased sensitivity to KBrO_3_ consistent with previously published data reporting that cells lacking MSH2 are more sensitive to methotrexate [17] or cytarabine and similar nucleoside analogs [23].

These results also provide a demonstration of both MSH2 dependent and independent toxicity pathways. In *Nth1* ^+/+^ cells, there was little evidence for MSH2 dependent pathways as MSH2 deficiency has no effect either on *t-*BOOH and KBrO_3_ toxicity (Table 1). However, in *Nth1* ^−/−^ cells, there was an MSH2 dependent pathway that acts on DNA damage, presumably oxidised pyrimidines induced by both *t*-BOOH and KBrO_3_. Similarly, MLH1 deficient cells are more resistant to *t-*BOOH than MLH1 proficient cells although the same level of DNA damage was observed in both cell lines [24]. In *Mpg* ^+/+^ cells, there was an MSH2 dependent pathway for KBrO_3_ but not *t-*BOOH induced toxicity (Table 1). Interestingly, Msh2 knockdown in *Mpg* ^−/−^ cells increased resistance to *t*-BOOH but increased sensitivity to KBrO_3_ suggesting that these agents induce different types (or levels) of adducts.

It is currently unclear why the loss of both MPG and MSH2 should differently alter the sensitivity to KBrO_3_ and *t*-BOOH. MPG removes alkyl DNA base products such as 7-methylguanine and 3-methyladenine [25] and also removes DNA toxic lesions induced by lipid peroxidation such as the etheno adduct 1,*N* ^6^-ethenoadenine [26]. However, MSH2 knockdown in *Mpg* ^−/−^ cells did not alter cellular toxicity induced by alkylating agents such as temozolomide and MMS, suggesting that alkyl adducts are not substrates for MSH2 [22]. Furthermore, alkyl DNA damage is unlikely to be induced by the agents used in this study. This then potentially implicates etheno or indeed other MPG substrates [27] as lesions that may be recognised by, the MutS homolog, MSH2 as MutS from *Escherichia coli* recognises exocyclic adducts arising from exposure to malondialdehyde [28]. Both *t*-BOOH [29] and KBrO_3_ [30] treatments can increase lipid peroxidation and potentially increase etheno DNA adducts, so that there does not seem to be a simple correlation between the persistence or absence of etheno DNA adducts and cellular response following Msh2 knockdown. However, this does not rule out the possibility that the different treatments used result in differing levels of etheno adducts and/or that KBrO_3_ results in a lesion whose persistence is directly cytotoxic irrespective of MSH2 function, whereas* t*-BOOH forms predominantly lesions whose toxicity is MSH2 dependent. The identity of these substrates is unclear. 

The extent of *Msh2* knockdown in the cell lines used was between ~80 and 90%. It is then possible that remaining MSH2 could have been sufficient to accomplish basic repair tasks, and though the results implicate DNA damage, the observed difference in sensitivity to *Msh2* knockdown may then have resulted from other mechanisms [31]. However, we have already shown that similar residual levels of MSH2 are not sufficient to prevent knockdown induced alterations in alkylating agent or 6-thioguanine induced cytotoxicity [22]. Other studies have also shown that a decrease in MSH2 expression of similar magnitude can have functional effects. For example, transfection of shRNA against *Msh2 *into CCD34-Lu/hTERT cells resulted in a 35–90% reduction in MSH2 protein level. Mean telomere shortening rate was significantly greater in those shMSH2 clones having between a 50 and 90% reduction in MSH2 protein level [32]. We cannot rule out, however, that residual MSH2 protein can be active in the repair of at least some of the types of DNA damage induced by the oxidising agents used particularly as the DNA damage induced is not fully characterised. 

Differences between clonogenic and MTT assays have been reported previously [33–35]; these differences are often compound and cell line specific and have been ascribed to differences in length of treatment allowing mechanistic differences between compounds to become apparent [33]. It is also possible that the MTT assay can reflect decreased mitochondrial function and not necessarily cell death [35]. In this study results for the MTT and clonogenic assays were consistent in three out of four cell lines following KBrO_3_ treatment. However, in contrast to the MTT results, the ability of the MEFs to proliferate in the presence of *t-*BOOH was largely unaffected by both the BER status and *Msh2 *status. The increased resistance to *t-*BOOH in BER deficient cells seen with the MTT assay was not observed in the clonogenic assay. It is possible that, in the BER deficient cells, the reduction in cell cycle arrest is temporary, and, as the cells accumulate more oxidative damage, from persistent exposure to *t-*BOOH, MMR-independent signals are sent for the cells to undergo apoptosis, such as from single- or double-strand DNA breaks, or that the damage is repaired by another DNA repair pathway such as nucleotide excision repair after the initial recognition by MMR. Therefore, in short-term MTT experiments a difference can be seen in cell survival, yet the cells that survive in the short-term are unable to proliferate in long-term experiments. In support of this, Chinese hamster B14 cells that survived treatment with *t-*BOOH were unable to proliferate after being replenished with fresh medium [29]. 

## 5. Conclusions

Results suggest the presence of MSH2 dependent and independent pathways to determine cellular toxicity induced by oxidising agents and a complex interaction between MMR and BER repair systems in determining cellular toxicity that is exposure dependent. A DNA repair gene-exposure interaction may then in humans help to determine susceptibility to ROS induced toxicity. 

## Figures and Tables

**Table 1:** Changes in resistance^a^ to *t*-BOOH and KBrO_3_ in cell lines as a result of *Msh2* knockdown in *Nth1* ^+/+^, *Nth1* ^−/−^, *Mpg* ^+/+^, and *Mpg* ^−/−^ MEFs.

Cell line	% *Msh2* gene silencing	*t*-BOOH		KBrO_3_	
		MTT	Clonogenic	MTT	Clonogenic
*Nth1* ^ +/+^	88%	—	~↑^‡^	—	—
*Nth1* ^−/−^	85%	↑***	—	↑***	↑*
*Mpg* ^ +/+^	85%	—	—	↑***	↑*
*Mpg* ^−/−^	79%	↑***	—	↓**	—

^a^Assessed by MTT and clonogenic assays.

*P ≤ 0.05; **P ≤ 0.01; ***P ≤ 0.001; ^‡^ *P* = 0.06.
